# 基于智能手机图像的移动反应界面电泳距离检测和分析

**DOI:** 10.3724/SP.J.1123.2023.06001

**Published:** 2023-09-08

**Authors:** Xinqiao SONG, Zehua GUO, Weiwen LIU, Genhan ZHA, Liuyin FAN, Chengxi CAO, Qiang ZHANG

**Affiliations:** 1.上海交通大学电子信息与电气工程学院, 上海 200240; 1. School of Electronic Information & Electrical Engineering, Shanghai Jiao Tong University, Shanghai 200240, China; 2.上海交通大学学生创新中心, 上海 200240; 2. Student Innovation Center, Shanghai Jiao Tong University, Shanghai 200240, China

**Keywords:** 电泳滴定, 血清标志物, 手机检测, 即时检测, electrophoretic titration, serum markers, smartphone detection, real-time detection

## Abstract

现有的电泳滴定(electrophoresis titration, ET)技术仍采用计算机进行数据处理和分析,其定量检测的即时性和便携性仍存在明显不足。针对这一问题,本文发展了一种基于智能手机的ET系统,实现了ET的即时性定量分析。该系统集成了三通道电泳滴定芯片与蓝牙通信功能,并设计了手机软件。通过该软件,不仅可以控制ET装置的电泳运行,还可以调用手机摄像头获取有色电泳界面,即时识别反应界面并显示定量检测结果。ET装置尺寸为10 cm×15 cm×2.5 cm,重300 g,可轻松手持,适用于现场检测。本文以人源血清总蛋白和尿酸(UA)为研究目标,分别使用基于聚丙烯酰胺凝胶的蛋白电泳酸碱滴定与基于琼脂糖凝胶的尿酸酶催化电泳滴定进行检测分析。用人血清白蛋白(HSA)标准品与尿酸标准品验证装置的性能,结果表明:HSA和UA的拟合优度(决定系数)分别为0.9959和0.9935,线性(或对数线性)范围分别为0.5~35.0 g/L和100~4000 μmol/L,检出限分别为0.05 g/L和50 μmol/L,相对标准偏差最大值分别为2.87%和3.21%,表明该系统具有较好的检测准确性和稳定性。选取了5位志愿者的血清样本,针对人源实际血样中的血清总蛋白含量和尿酸含量进行检测,并与医院临床检测所使用方法的检测结果进行对比,检测相对误差分别≤6.03%和6.21%,证明本文提出的检测系统是一种具有综合性检测潜力的通用平台,具有临床应用价值与现场检测潜力。

## Abstract (en)

Electrophoresis titration (ET) based on the moving reaction boundary (MRB) theory can detect the analyte contents in different samples by converting content signals into distance signals. However, this technique is only suitable for on-site qualitative testing, and accurate quantification relies on complex optical equipment and computers. Hence, applying this method to real-time point-of-care testing (POCT) is challenging.

In this study, we developed a smartphone-based ET system based on a visual technique to achieve real-time quantitative detection. First, we developed a portable quantitative ET device that can connect to a smartphone; this device consisted of five components, namely, an ET chip, a power module, a microcontroller, a liquid crystal display screen, and a Bluetooth module. The device measured 10 cm×15 cm×2.5 cm, weighed 300 g, and was easy to hold. Thus, it is suitable for on-site testing with a run time of only 2-4 min. An assistant mobile software program was also developed to control the device and perform ET. The colored electrophoresis boundary can be captured using the smartphone camera, and quantitative detection results can be obtained in real time. Second, we proposed a quantitative algorithm based on ET channels. The software was used to recognize the boundary migration distance of three channels, a standard curve based on two given contents of the standards was established using the two-point method, and the content of the test sample was calculated. Human serum albumin (HSA) and uric acid (UA) were used as a model protein and biosample, respectively, to test the performance of the detection system. For HSA detection, different HSA solutions were mixed with a polyacrylamide gel (PAG) stock solution, phenolphthalein was added as an indicator, and sodium persulfate and tetramethyl ethylenediamine (TEMED) were used to promote polymerization to form a gel. For UA detection, agarose gel was filled into the ET channel, the UA sample, urate oxidase, and leucomalachite green were added into the anode cell and incubated for 20 min. ET was then performed. The fitting goodness (*R*^2^) values of HSA and UA were 0.9959 and 0.9935, respectively, with a linear range of 0.5-35.0 g/L and a log-linear range of 100-4000 μmol/L. The limits of detection for HSA and UA were 0.05 g/L and 50 μmol/L, respectively, and the corresponding relative standard deviations (RSDs) were not greater than 2.87% and 3.21%, respectively. These results demonstrate that the detection system has good accuracy and sensitivity.

Clinical samples collected from healthy volunteers were used as target blood samples, and the developed system was used to measure serum total protein and UA levels. Serum samples from five volunteers were selected, standard curves of total serum protein and UA were established, and the test results were compared with hospital standard testing results. The relative errors for serum total protein and UA were less than 6.03% and 6.21%, respectively, and the corresponding RSDs were less than 3.72% and 5.84%, respectively. These findings verify the accuracy and reliability of the proposed detection system. The smartphone-based ET detection system introduced in this paper presents several advantages. First, it enables the portable real-time detection of total serum protein and UA. Second, compared with traditional ET strategies based on colored boundaries, it does not rely on optical detection equipment or computers to obtain quantitative detection results; as such, it can reduce the complexity of the operation and provide portability and real-time metrics. Third, the detection of two biomarkers, serum total protein and UA, is achieved on the same device, thereby improving the multitarget detection potential of the ET method. These advantages render the developed method a promising detection platform for clinical applications and real-time POCT.

基于移动反应界面(moving reaction boundary, MRB)^[1,2]^的电泳滴定(electrophoresis titration, ET)^[3,4]^可以实现将检测物质的浓度信号转化为距离信号,以检测不同的生化标志物,如有机小分子^[5⇓-7]^、核酸^[8]^、蛋白质^[9⇓-11]^等。经典的MRB技术利用酸碱指示剂或有色化合物将电泳过程中的反应界面以有色界面的形式呈现,实现对低浓度目标物含量的可视检测,但其依赖于稳定的高压电源及大型且昂贵的检测设备^[12⇓⇓⇓-16]^,难以应用于现场即时检测(point of care testing, POCT)。

为了解决上述问题,需要进一步推进ET装置的小型化与便携化。Wang等^[9⇓-11]^开发了基于ET芯片的电泳滴定方法,首次将可视化的电泳滴定应用于蛋白检测。Li等^[6]^实现了三聚氰胺的ET检测方法,用于检测乳制品中掺杂的三聚氰胺。Khan等^[5]^提出了基于ET芯片的尿酸(uric acid, UA)检测方法,通过尿酸酶催化反应实现了UA的可视化检测。Wang等^[17]^基于先前的工作提出了双内标-电泳滴定(double inner standard plot model of electrophoresis titration, DISP-ET)检测模型,可以实现乳品总蛋白含量的现场定性检测。这些方法实现了ET检测的小型化,可以快速实现定性分析,但是后续定量分析仍需光学检测设备以及计算机软件,难以实现即时、精确的定量检测。

针对上述问题,本工作开发了一种基于智能手机的移动反应界面电泳距离传感检测系统,实现了ET方法的便携式即时定量分析。该系统由ET装置和智能手机组成,两者通过蓝牙通信,并在手机端设计了相应的安卓软件。此软件可以控制ET装置的电泳运行,期间通过手机摄像头获取电泳结果,最终经程序分析给出定量结果。本文以血清总蛋白和尿酸为研究目标验证该系统的性能,对人源血清白蛋白(human serum albumin, HSA)标准品和尿酸标准品进行了检测分析,并对从医院获得的真实血清样本的总蛋白含量和尿酸含量进行了检测,验证了所开发装置的有效性和准确性,证明本文提出的检测系统是一种具有综合性检测潜力的通用平台,在临床分析和现场检测中有着潜在的应用价值。

## 1 实验部分

### 1.1 仪器与试剂

高速离心机(贝克曼Allegra X-12,美国)用于从全血中分离血清样本。电子天平(梅特勒托利多ML204/02,精度为0.1 mg,瑞士)用于样品称量。超纯水系统(SG Water公司,德国)用于生产去离子水。智能手机(华为Mate9,中国)用于控制检测过程及分析检测结果。

氯化钾和氢氧化钠购自凌峰化学试剂有限公司;丙烯酰胺(acrylamide)、HSA、UA、尿酸氧化酶(urate oxidase)和辣根过氧化物酶(horseradish peroxidase, HRP)购自上海麦克林生化科技有限公司;双丙烯酰胺(bis-acrylamide)购自国药化学试剂有限公司;酚酞(phenolphthalein)和过硫酸铵(ammonium persulfate, APS)购自上海化学试剂有限公司;四甲基乙二胺(tetramethyl ethylenediamine, TEMED)购自生工生物工程有限公司;隐色孔雀石绿(leucomalachite green, LMG)购自上海西格玛奥德里奇贸易有限公司;琼脂糖(agarose, AG)购自上海贝晶生物技术有限公司。所有化学试剂均为分析纯。

实际血样取自上海市第六人民医院(临港院区),本研究已通过上海交通大学附属胸科医院临床研究伦理委员会审批,批准号:KS2011。

### 1.2 溶液制备与实验操作

血液样本前处理 使用乙二胺四乙酸(EDTA)抗凝管采集静脉血5 mL,将血液在离心机中以4000 r/min的速度离心5 min后,提取上层清液,然后将上清液加入超滤浓缩离心管以5000 r/min的速度离心20 min,样本体积浓缩到原体积1/3左右,吸取并弃去滤液,向超滤管中加入与滤液等体积的超纯水,保存在-20 ℃冰箱中备用。

总蛋白检测 聚丙烯酰胺凝胶(polyacrylamide gel, PAG)母液:19.4 g的丙烯酰胺和0.6 g双丙烯酰胺溶于水,定容至100 mL;阳极液:100 mmol/L KCl;阴极液:100 mmol/L NaOH; HSA母液:100 g/L的HSA溶液,储存在4 ℃的冰箱备用。固定液制备:PAG母液与不同浓度的HSA溶液(将HSA母液稀释成30、45、60、75、90 g/L的HSA溶液)混合,加入KCl和酚酞,使得溶液内KCl浓度为0.1 mmol/L,酚酞质量分数为0.1%,依次加入APS和TEMED促凝。将固定液填充到ET芯片的3条通道中形成凝胶。凝胶固化后分别加入阳极液和阴极液,根据预先设置的电泳时间进行电泳,过程中随时通过软件调用手机摄像头获取有色电泳界面并经程序计算得出检测值。

尿酸检测 琼脂糖凝胶母液:将0.1 g的琼脂糖、0.5 mL的2 mol/L的KCl和0.1 mL的0.1 mol/L的乙酸钠混合,加水定容至10 mL;阳极液:含有不同浓度(100、150、250、400和1000 μmol/L) UA、 0.5 mg/mL尿酸酶、1 mmol/L LMG和10 μg/mL HRP的水溶液;阴极液:含有100 mmol/L KCl和100 μmol/L乙酸钠的水溶液。将琼脂糖加热溶解后注入ET芯片3条通道中形成凝胶,凝胶固化后分别加入阳极液和阴极液,根据预先设置的电泳时间进行电泳,过程中随时通过软件调用手机摄像头获取有色电泳界面并经程序计算得出检测值。

临床样本测定方法 医院所使用的人源血清白蛋白和尿酸检测方法为双缩脲法和酶偶联法,具体方法见参考文献^[18,19]^。

### 1.3 基于智能手机的ET检测系统

图1a为便携式ET装置模型示意图,装置尺寸为10 cm×15 cm×2.5 cm,重300 g,可轻松手持,适用于现场检测。智能手机及开发的软件界面如图1b所示,基于智能手机ET装置的结构图如图1c所示。用户通过手机软件发送控制信息,装置中的蓝牙模块接收后控制电源模块中的升压模块为ET芯片提供24 V电压,以控制电泳的运行,或通过液晶触摸显示屏控制电泳过程。装置上提供电泳拍摄窗口,在电泳运行过程中,通过手机软件调用手机摄像头,拍摄窗口内电泳结果,并通过内置算法给出电泳检测物质及定量检测结果。手机软件是基于安卓系统开发的一款应用程序,主要包括蓝牙连接模块、数据分析模块和数据展示模块。蓝牙连接模块负责与装置通信,控制装置电泳的进行。数据分析模块负责处理电泳结果。最后电泳结果处理图及定量检测结果通过数据展示模块展示在手机屏幕上。

**图 1:**
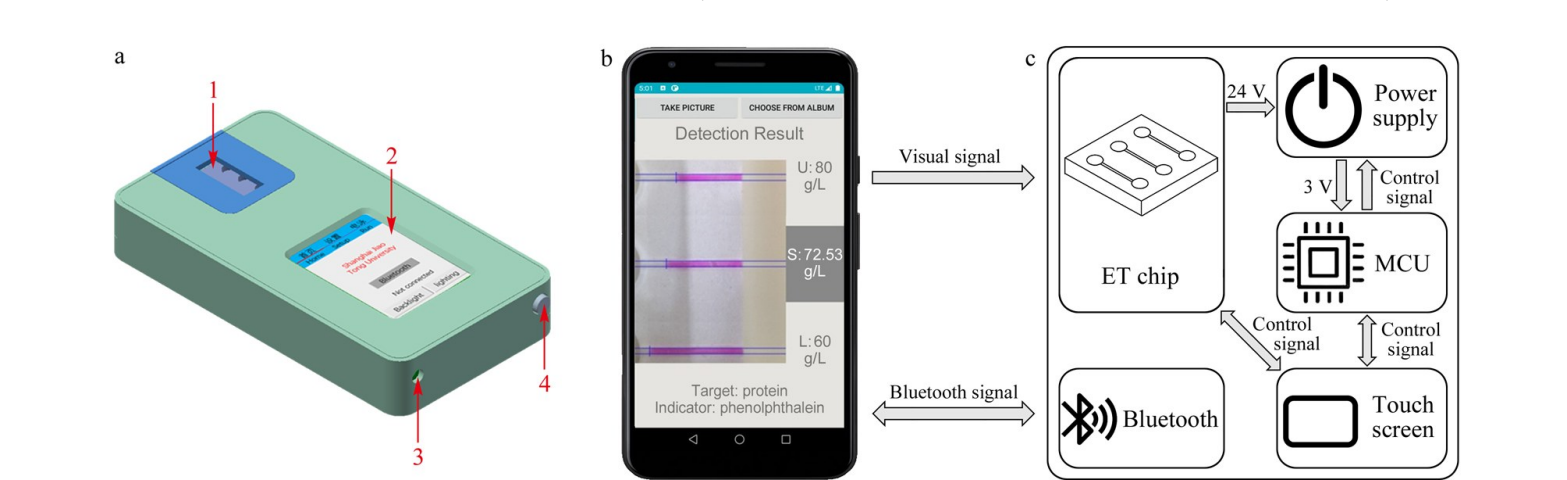
ET检测系统示意图

### 1.4 浓度-距离传感和定量计算方法

基于酸碱滴定的蛋白ET原理 根据MRB蛋白电泳滴定原理^[9⇓-11,17]^,在电场的作用下,蛋白ET的运行时间*t*与MRB迁移距离*D*呈线性关系,运行时间越长,界面迁移距离越长。在运行时间相同时,界面迁移距离与蛋白质质量浓度*C*_HSA_呈线性关系,蛋白质浓度越高,界面迁移距离越短。在三通道芯片中的两个通道内固定已知浓度的蛋白标准品,在剩下的一个通道内固定未知浓度的待测蛋白样本,在相同电压下运行相同时间,测量出3条通道中的界面迁移距离。为了减小现场复杂环境因素对标准曲线建立的影响,提高检测系统的即时性,本文通过两个蛋白标准品的浓度和界面迁移距离建立标准曲线,再将待测蛋白样本的界面迁移距离带入标准曲线,得出待测蛋白样本的浓度。

基于酶促反应的尿酸ET原理 根据MRB酶促尿酸电泳滴定原理^[5]^,在电场的作用下,尿酸ET的运行时间*t*与MRB迁移距离*D*呈线性关系,运行时间越长,界面迁移距离越长。与蛋白ET不同的是,在运行时间相同时,界面迁移距离与尿酸浓度*c*值的对数呈线性关系,且尿酸浓度越高,界面迁移距离越长。因此,不同于蛋白电泳,检测尿酸是通过两个尿酸标准品浓度的对数和界面迁移距离建立标准曲线,再将待测尿酸样本的界面迁移距离带入标准曲线,得出待测尿酸样本浓度的对数,换算后得出待测尿酸样本的浓度。

手机识别与定量方法 使用手机摄像头在自然光下采集ET图像,拍摄时保证3条通道的反应界面都处在电泳拍摄窗口内,并尽量将图片边框对准装置上的电泳拍摄窗口,使得有色通道在图片中基本处于水平位置。ET运行时间较短时,三通道的界面差异较小,建立标准曲线进行检测会造成较大的误差,在反应界面运行到电泳拍摄窗口中可以保证界面迁移时间大于1~2 min,界面迁移距离大于2 mm,此时建立标准曲线检测误差较小。采集到ET图像后,在手机上对图像进行了一系列处理,具体操作流程见图2。首先对图像做灰度化和高斯滤波的预处理,得到平滑的灰度图像。接下来使用Canny边缘检测算法获取3条通道的边缘位置。找到3条通道后,通过通道内横向梯度变化率找到反应界面。识别通道内指示剂的颜色,在本文使用的体系中,品红色为酚酞在碱性环境下的颜色,蓝绿色为MG^+^(由LMG生成)的颜色,分别对应蛋白与尿酸的检测。针对不同的检测物质,通过已知的两个标准品浓度及其界面迁移距离建立标准曲线(蛋白对应线性曲线,尿酸对应对数线性曲线),再通过待测样本的边界迁移距离计算出待测样本浓度,显示在手机屏幕上。

**图 2:**
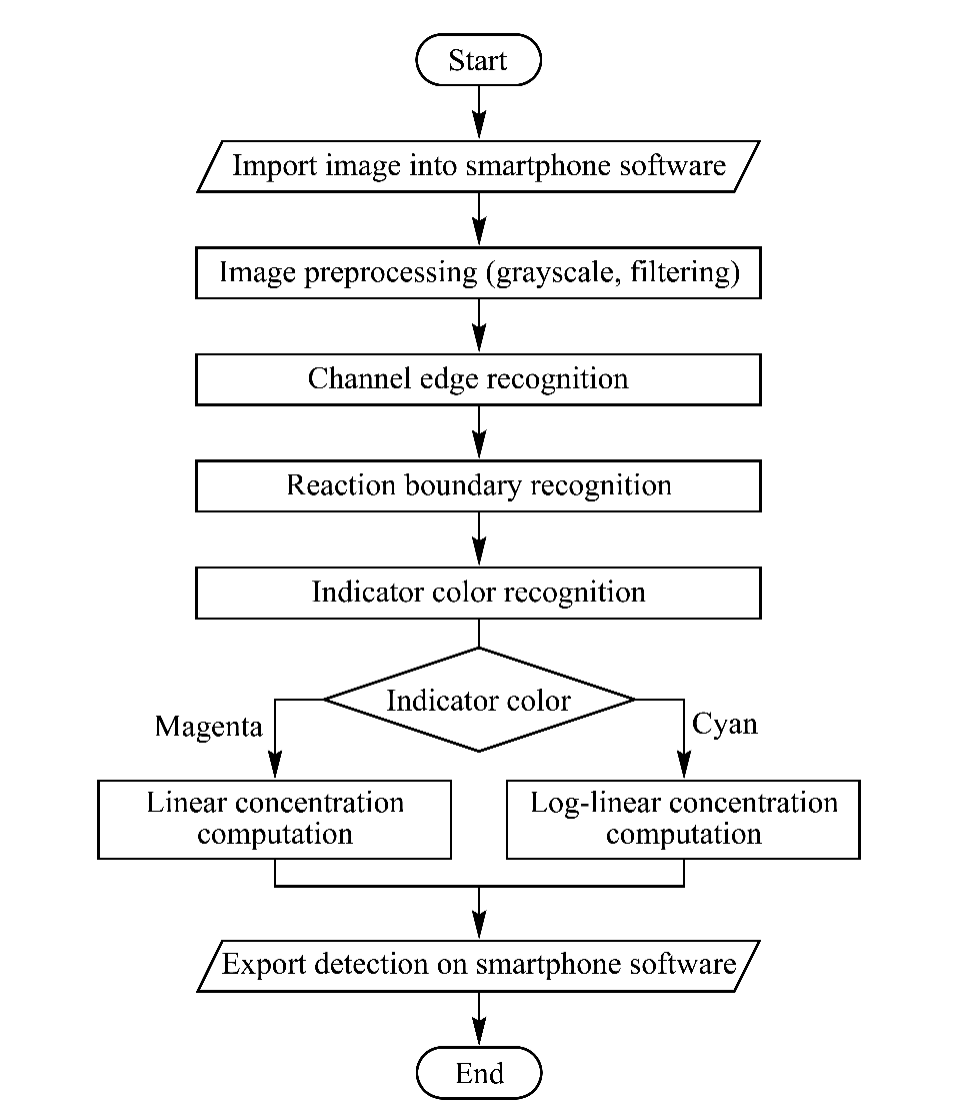
ET结果识别和定量方法流程图

## 2 结果与讨论

### 2.1 HSA及尿酸的测定

血清总蛋白中HSA的占比最高,主要起维持渗透压、pH缓冲、载体和营养作用,其检测对恶性肿瘤、营养及吸收障碍、肝硬化、肾病综合征等多种疾病的临床诊断具有重要意义^[20⇓-22]^。本文采用HSA对系统的性能进行初步验证。图3a、b显示了ET中MRB迁移距离与HSA含量的实验结果。如图3a所示,HSA含量越高,在给定运行时间内MRB运动距离越短。在选定的5种浓度中,界面迁移距离和运行时间的拟合优度(以决定系数(*R*^2^)表示)为0.9983~0.9994。根据图3a中120 s的界面迁移距离,建立了如图3b所示的界面迁移距离与HSA含量之间的关系。可以看出,界面迁移距离与HSA含量之间具有良好的线性关系(*R*^2^=0.9959)。

**图 3:**
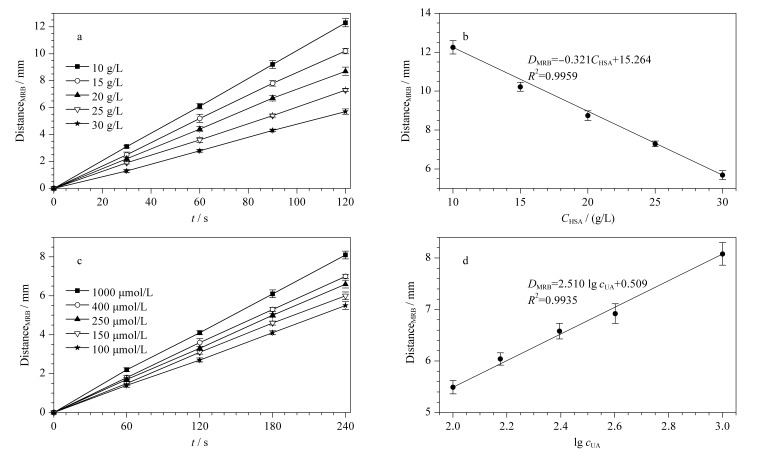
采用HSA和UA对于检测系统进行性能测试的结果图(*n*=3)

UA是嘌呤代谢排泄到血液和尿液中的主要终产物,血液中尿酸的测定对痛风、肾衰竭、慢性肾病、高尿酸血症等多种疾病的临床诊断具有重要意义^[22,23]^。图3c、d显示了ET中MRB迁移距离与UA含量的实验结果。如图3c所示,UA含量越高,在给定运行时间内MRB迁移距离越长。在选定的5种浓度中,界面迁移距离和运行时间的拟合优度*R*^2^为0.9970~0.9991。根据图3c中240 s的界面迁移距离,建立了如图3d所示的界面迁移距离与UA含量之间的关系。可以看出,界面迁移距离与UA含量之间具有良好的对数线性关系(*R*^2^=0.9935)。

表1展示了ET检测HSA与UA的线性范围、检出限及相对标准偏差(RSD),其中检出限是根据手机软件对界面迁移距离的最小识别尺度与同期空白对照所得出的最小标准样品浓度确定,RSD为图3中HSA和UA样本5个含量水平的RSD最大值。从表1中可以看出,HSA的线性范围为0.5~35.0 g/L,而正常人血清总蛋白含量为60~80 g/L,血清经稀释后,ET具有测量血清总蛋白含量的能力。UA的线性范围为100~4000 μmol/L,正常人血尿酸含量为208~428 μmol/L,包含在ET检测范围内。同时,ET方法具有较低的检出限(0.05 g/L和50 μmol/L)以及较好的重复性(RSD为2.87%和3.21%)。

**表 1:** HSA与UA的线性范围、LOD及RSD

Analyte	Linear range	LOD	RSD/%(n=3)
HSA	0.5-35.0 g/L	0.05 g/L	2.87
UA	100-4000 μmol/L	50 μmol/L	3.21

HSA: human serum albumin; UA: uric acid. LOD: limit of detection; RSD: relative standard deviation.

### 2.2 实际样本分析

为了验证ET检测系统对实际血清样本总蛋白(TP)含量和UA含量检测的有效性,首先选取志愿者血样,该血样血清总蛋白含量由医院测得为68.4 g/L,UA含量为391 μmol/L (1号样本)。进行梯度稀释后得到血清TP质量浓度为9.8、17.1、22.8、27.4和34.2 g/L的样本,用于血清总蛋白的测量。另取该血样进行梯度稀释后得到UA浓度为97.8、156.4、260.7和391.0 μmol/L的样本,加标得到浓度为991.0 μmol/L的样本,用于UA的测量。图4a和4c中,界面迁移距离和运行时间的拟合优度与标准品检测类似,*R*^2^为0.9942~0.9989,表明检测系统在血清总蛋白和血尿酸的检测上具有良好的检测性能。图4b和4d分别为运行时间为120 s和240 s时的标准曲线,与HSA和UA标准品的检测相比,其相同时间内界面迁移距离明显缩短,推测原因为血清中的其他物质对ET有一定的阻滞作用。

**图 4:**
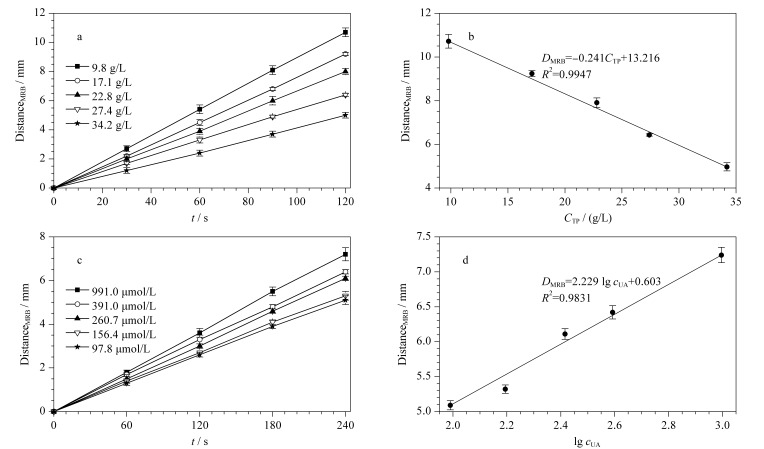
采用实际血样中总蛋白和尿酸对于检测系统进行性能测试的结果图(*n*=3)

本文选取了5位志愿者的血清样本进行ET检测,检测总蛋白时将血清稀释3倍,检测尿酸时无需稀释。智能手机得出的检测结果如图5a和5b所示,可以看到,界面中给出了检测所用的指示剂、待测物质以及检测结果。将该检测结果与医院所提供的检测结果比对(见表2和表3),可以看出,ET方法的血清总蛋白检测结果与医院数据的相对偏差≤6.03%,ET方法的RSD为1.40%~3.72%;尿酸检测的相对偏差≤6.21%,RSD为2.63%~5.84%。

**图 5:**
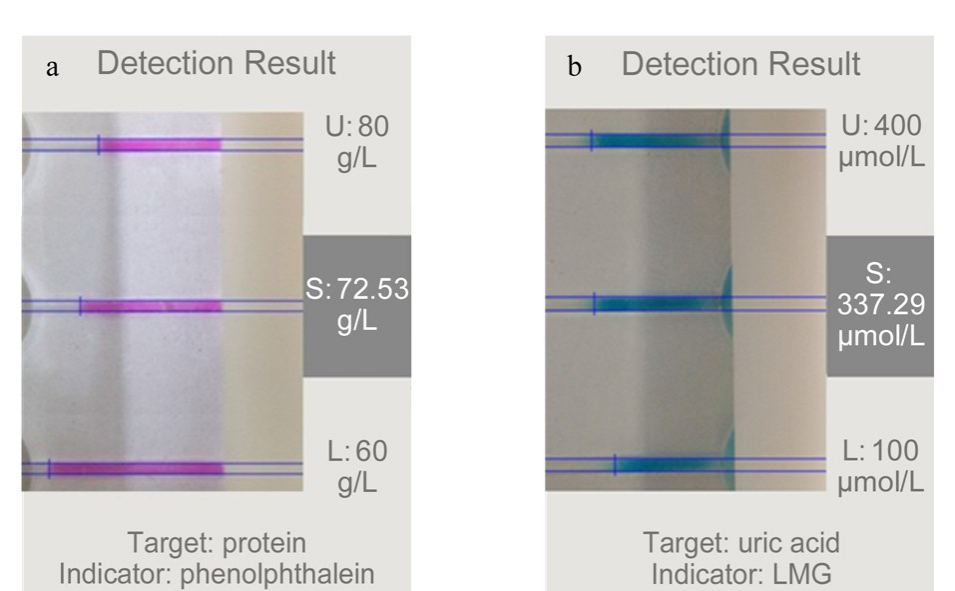
(a)血清总蛋白和(b)尿酸实际检测结果图

**表 2:** 临床血清样本总蛋白检测结果

Serum sample	Hospital detection result/(g/L)	ET method/ (g/L)	Relative error/%	RSD of ET method/ %(n=3)
1	68.4	71.39	4.37	1.55
2	73.7	78.14	6.03	1.48
3	61.8	64.96	5.12	2.80
4	48.5	49.88	2.85	3.72
5	51.8	54.22	4.68	3.49

**表 3:** 临床血清样本尿酸检测结果

Serum sample	Hospital detection results/(μmol/L)	ET method/ (μmol/L)	Relative error/%	RSD of ET method/ %(n=3)
1	391	401.1	2.58	3.18
2	327	337.2	3.12	2.96
3	380	403.6	6.21	4.25
4	156	156.7	0.45	5.84
5	163	172.5	5.83	2.63

结果证明该方法具有较好的准确性和稳定性,可应用于实际血清样本的即时定量检测。

## 3 结论

本文发展了一种基于智能手机的便携式ET系统,并将其用于血清总蛋白和血尿酸的检测。通过自主研发的手机软件不仅可以无线控制ET装置的电泳运行,还可以即时拍摄并定量计算出电泳结果,进一步提高了ET系统的便携性。为验证系统的性能,使用HSA和UA标准品进行测试,并进行了血清总蛋白和UA这两种不同的血生化指标的检测。实验结果表明,该系统具有线性好、准确性高、重复性好等优点,尤其是具有即时性和便携性,能够完成现场定量检测任务,是一种具有综合性检测潜力的通用平台,具有临床应用价值与现场检测潜力。
